# Transversus abdominis release (TAR) for ventral hernia repair: open or robotic? Short-term outcomes from a systematic review with meta-analysis

**DOI:** 10.1007/s10029-021-02487-5

**Published:** 2021-09-07

**Authors:** U. Bracale, F. Corcione, D. Neola, S. Castiglioni, G. Cavallaro, C. Stabilini, E. Botteri, M. Sodo, N. Imperatore, R. Peltrini

**Affiliations:** 1 Department of General and Specialistic Surgeries, Federico II University Hospital, Naples, Italy; 2 Department of Public Health, University of Naples Federico II, Naples, Italy; 3 Department of Medical, Oral and Biotechnological Sciences, University G. D’Annunzio Chieti-Pescara, Pescara, Italy; 4 Department of Surgery “P. Valdoni”, University of Rome “La Sapienza”, Rome, Italy; 5 Department of Surgical Sciences, University of Genoa, Policlinico San Martino IRCCS, Genoa, Italy; 6 General Surgery, ASST Spedali Civili Di Brescia, Brescia, Italy; 7 Department of Clinical Medicine and Surgery, University of Naples Federico II, Naples, Italy; 8 Department of Public Health, School of Medicine and Surgery, University of Naples Federico II, Via Pansini 5, 80131 Naples, Italy

**Keywords:** Transversus abdominis release, Robotic TAR, Ventral hernia repair

## Abstract

**Purpose:**

To compare early postoperative outcomes after transversus abdominis release (TAR) for ventral hernia repair with open (oTAR) and robotic (rTAR) approach.

**Methods:**

A systematic search of PubMed/MEDLINE, EMBASE, SCOPUS and Web of Science databases was conducted to identify comparative studies until October 2020. A meta-analysis of postoperative short-term outcomes was performed including complications rate, operative time, length of stay, surgical site infection (SSI), surgical site occurrence (SSO), SSO requiring intervention (SSOPI), systemic complications, readmission, and reoperation rates as measure outcomes.

**Results:**

Six retrospective studies were included in the analysis with a total of 831 patients who underwent rTAR (*n* = 237) and oTAR (*n* = 594). Robotic TAR was associated with lower risk of complications rate (9.3 vs 20.7%, OR 0.358, 95% CI 0.218–0.589, *p* < 0.001), lower risk of developing SSO (5.3 vs 11.5%, OR 0.669, 95% CI 0.307–1.458, *p* = 0.02), lower risk of developing systemic complications (6.3 vs 26.5%, OR 0.208, 95% CI 0.100–0.433, *p* < 0.001), shorter hospital stay (SMD − 4.409, 95% CI − 6.000 to − 2.818, *p* < 0.001) but longer operative time (SMD 53.115, 95% CI 30.236–75.993, *p* < 0.01) compared with oTAR. There was no statistically significant difference in terms of SSI, SSOPI, readmission, and reoperation rates.

**Conclusion:**

Robotic TAR improves recovery by adding the benefits of minimally invasive procedures when compared to open surgery. Although postoperative complications appear to decrease with a robotic approach, further studies are needed to support the real long-term and cost-effective advantages.

## Introduction

Incisional hernia is one of the most common complications following abdominal surgery, with a reported incidence of 3–13% [1]. Ventral hernia repair (VHR) including Primary Ventral Hernia (PVH) and Incisional Hernia (IH), with up to 350,000 procedures performed annually, remains one of the most common procedures performed by general surgeons, amounting over $3.4 billion dollars in health costs [2].

There has been a recent trend in hernia surgery to reconstruct abdominal wall defects by reestablishing the fascial edges to their normal anatomic position, through a retromuscular approach. The Rives-Stoppa technique has been adopted by the Americas Hernia Society as the gold standard procedure for VHR with a recurrence less than 5% [3, 4]. Transversus abdominis release (TAR) was introduced in 2012 as a modification of the Rives-Stoppa technique [4], and it has become one of the procedures of choice in case of wide and complex ventral hernias. Respect to other types of component separation, it seems to decrease the risk of wound morbidity and hernia recurrence. In the last years, some surgeons have begun to investigate the role of the mini-invasive approach in retromuscular repairs. While TAR has been described laparoscopically [5], it is rarely performed in this manner due to several limitations as ergonomic, mechanical, and technical.

With advances in robotic surgery, to combine the benefits of minimally invasive approach and TAR, robotic retromuscular repairs are increasingly being used to treat complex ventral hernias. Some recent studies suggested that robotic approach seems to have some benefits despite an hypothetical increase of health-system costs. Therefore, the purpose of this study was to compare clinical outcomes, through a systematic review with meta-analysis, of robotic TAR (rTAR) and Open TAR (oTAR) for VHR.

## Materials and methods

### Literature search and selection of primary studies

The strategy for building the evidence base for the assessment of the outcomes of rTAR versus oTAR was performed with a systematic review of the existing evidence in the literature, conducted in accordance with the preferred reporting items for systematic reviews and meta-analyses (PRISMA) guidelines [6].

The systematic literature review was performed in PubMed/MEDLINE, EMBASE, SCOPUS and Cochrane databases to identify studies that compared outcomes of rTAR vs oTAR reinforcement from the beginning of indexing for each database till Sep 1, 2020. Bibliographic review of selected articles was assessed as secondary sources for full-length articles of studies. A literature search was performed and verified by 2 independent reviewers (R.P. and N.I.) using the following index terms: “transversus abdominis release” AND “robotic” OR “open” AND “abdominal wall reconstruction” OR “hernia”.

### Eligibility criteria

Two reviewers (R.P. and N.I.) independently evaluated all the studies retrieved according to the eligibility criteria and any differences between the datasets were resolved by discussion. Studies were included if they met all of the following criteria: (1) case–control studies, randomized controlled trial (RCT), prospective or retrospective studies directly comparing rTAR and oTAR; (2) original studies published in a peer-reviewed journal; (3) studies involving adult patients (aged > 18 years). We excluded the articles if there was no sufficient documentation on—or no possibility to calculate—the percentage of postoperative complications (primary endpoint), if they were in languages other than English, if they were focused on pediatric patients. Narrative reviews, duplicate publications and editorials were also excluded.

### Data extraction and management

Data were extracted independently and entered into standardized Excel spreadsheets (Microsoft Inc., Redmond, Washington, USA). Any disagreements were resolved through discussion. The following data were extracted from each study: first author, year of publication, study design, sample size, type of TAR (robotic or open), age, gender, BMI, ASA status, operative time (minutes), length of stay (LOS, days), post-operative complications, number of patients developing surgical site infection (SSI), surgical site occurrence (SSO), SSO requiring intervention, number of systemic complications, percentage of readmission and reoperation.

Primary study outcome was the assessment of post-operative complications rate in the two groups (rTAR vs oTAR) at the end of follow-up for each included study. Furthermore, secondary outcomes included the evaluation of differences in: operative time, SSI development, SSO, SSO requiring intervention (SSOPI), number of systemic complications, need for readmission and reoperation and LOS.

### Statistical analysis

Statistical analyses were performed by using Comprehensive Meta-analysis Software version 3.0 (Biostat, Englewood, New Jersey, USA).

Heterogeneity was assessed by using Chi-squared statistics and I2 measure of inconsistency. The quality of the analyzed studies and publication bias was evaluated by two reviewers (R.P. and N.I) in consensus using a quality assessment tool for diagnostic accuracy studies (QUADAS-2) [7]. The risk of publication bias and concerns regarding the applicability of studies were then assessed by visually inspecting QUADAS-2 plots.

The meta-analysis was conducted using a fixed-effect model in the case of non-significant heterogeneity (*p* > 0.1), and a random effect model (DerSimonian–Laird method) when significant heterogeneity was present (*p* < 0.1). Corresponding forest plots were constructed for the pooled estimates of these outcomes and weight of individual studies are represented by the size of individual squares. The odds ratio (OR) was assessed for dichotomous outcomes, while standardized mean difference (SMD) with 95% confidence interval (CI) was estimated for continuous outcomes.

Furthermore, a random effect meta-regression was performed to evaluate possible patient (age, gender, comorbidities such as diabetes, BMI, respiratory diseases) or disease (type of hernia, hernia dimensions) or technical (use and type of mesh) variables able to impact upon the outcomes. Similarly, a random effect meta-regression was used to evaluate how the length of follow-up affected the primary outcome.

A *p* value < 0.05 was considered statistically significant for all outcomes.

## Results

**Fig. 1:**
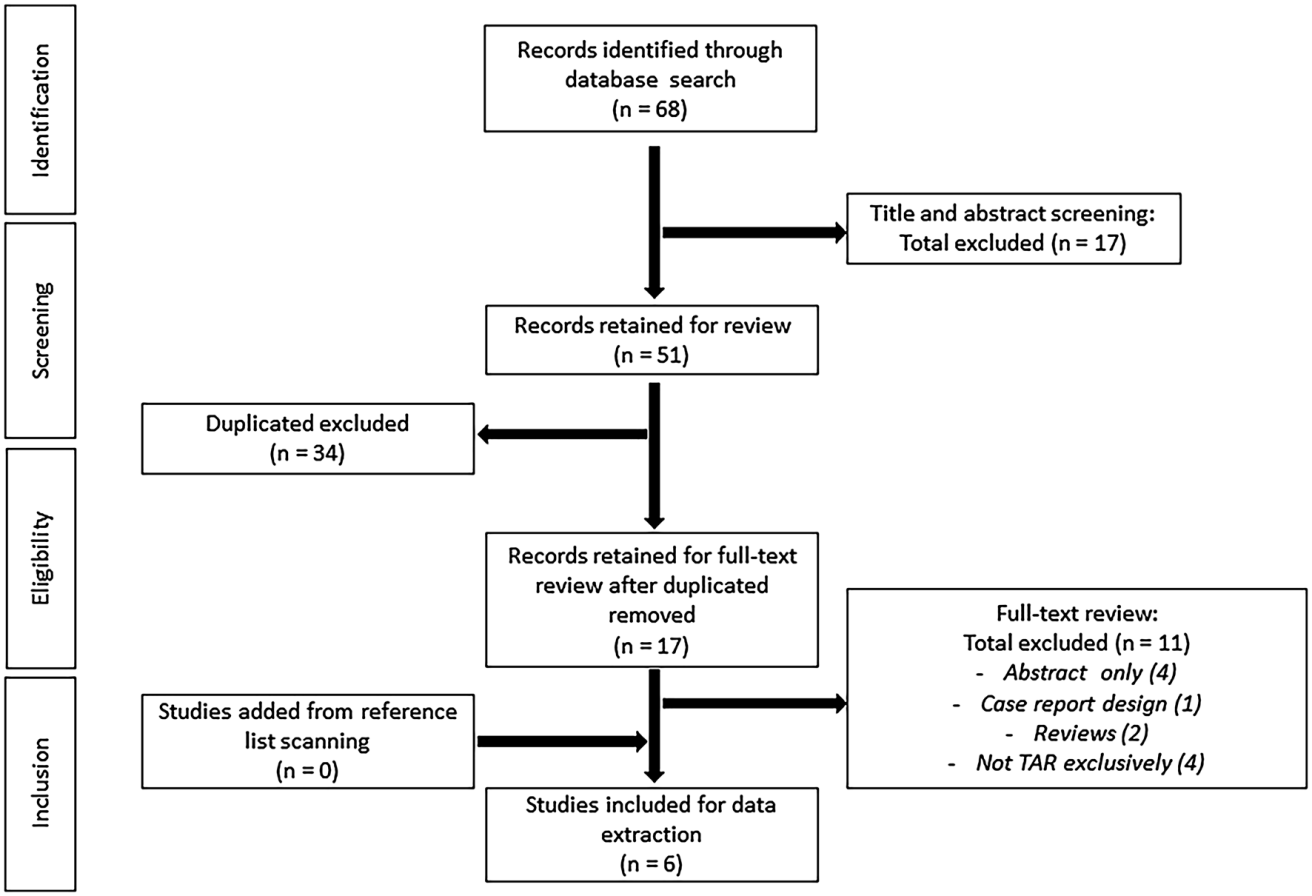
Flow diagram of the search strategy and selection of studies included in the meta-analysis

Figure 1 shows the PRISMA flow diagram of the literature selection process. The search strategy identified a total of 68 publications in the initial search. After the screening of title and abstract and removal of duplicates, 17 articles were selected for further review. After exclusion of 11 articles based on exclusion criteria, 6 studies were included in the meta-analysis [8–13].

In accordance with the inclusion criteria, all studies were retrospective case–control studies based on prospective maintained database.

Finally, a total of 831 patients who underwent TAR with a robotic approach (*n* = 237) or open approach (*n* = 594) were included in the meta-analysis. Table 1 shows the baseline characteristics of the studies included.

**Table 1:** Details of studies selected for meta-analysis

Authors	Study period	Study design	Sample size	Study group		Mean hernia width/length (cm)		Hybrid robotic repair	Robotic platform	Follow-up (days)
				rTAR	oTAR	rTAR	oTAR			
Abdu et al. [8]	2016–2018	Retro–PSM	380	95	285	12/19	12/19	Yes	NR	30
Bittner et al. [9]	2015–2016	Retro	102	26	76	12.3 ± 3/18.5 ± 5.1	13.7 ± 5.9/17.1 ± 7.1	No	Da Vinci Si + Xi	90
Dauser et al. [10]	2017–2019	Retro	26	16	10	NR	NR	No	Da Vinci Si	30
Halka et al. [11]	2013–2017	Retro	183	49	134	38.8 ± 6.7/41.6 ± 6.1	37.4 ± 8.3/42.2 ± 9.1	Yes	Da Vinci Xi	30
Martin-del-Campo et al. [12]	2015–2016	Retro	114	38	76	13.5 ± 4.5/NR	13.5 ± 4.5/NR	No	NR	NR
Reeves et al. [13]	2018–2020	Retro	26	13	13	NR	NR	No	Da Vinci Xi	30

*rTAR* robotic transversus abdominis release, *oTAR* open transversus abdominis release, *NR* not reported, *Retro* retrospective study, *PSM* propensity score matching

### Quality of studies and risk of bias

**Fig. 2:**
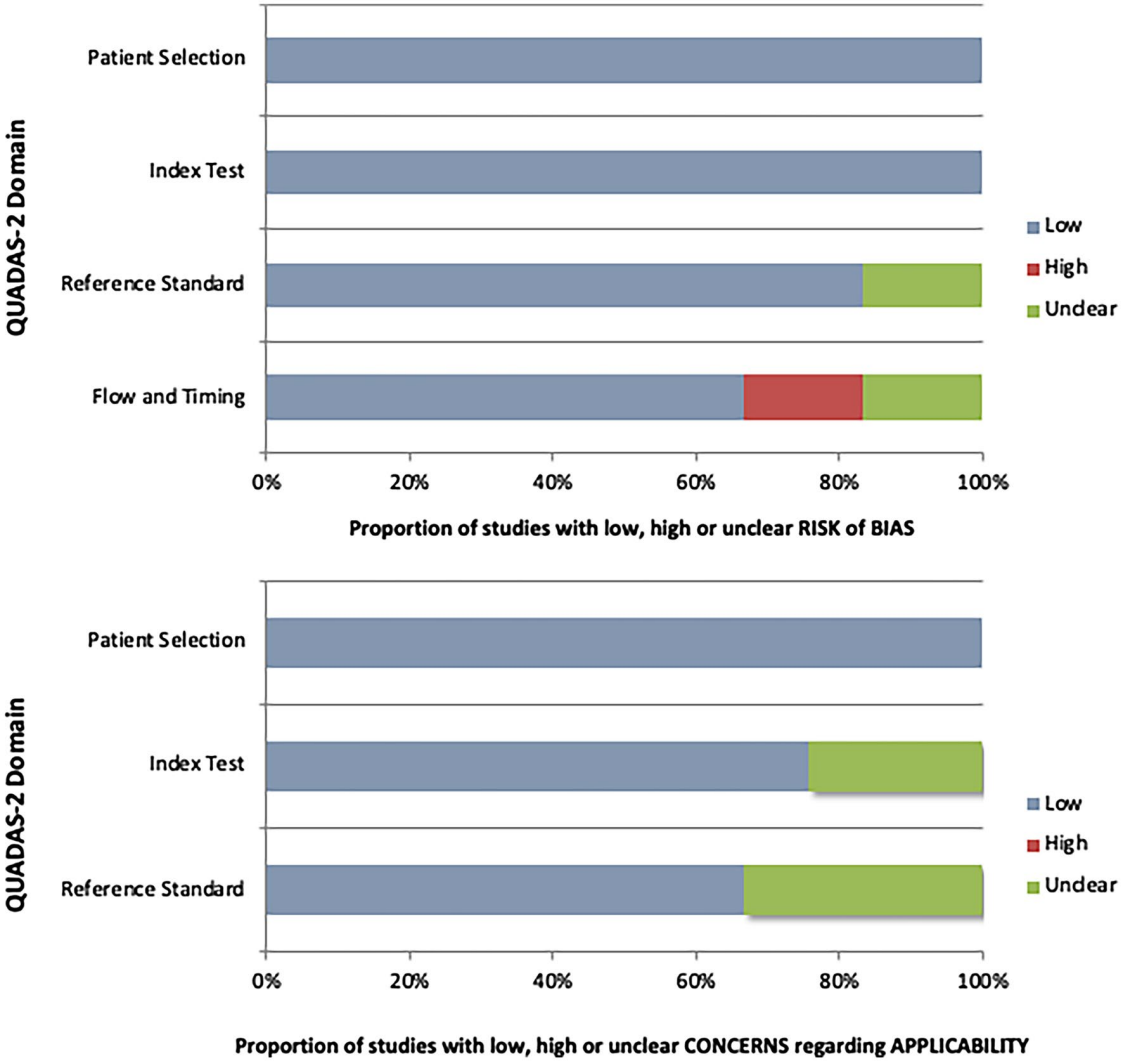
QUADAS-2 studies evaluation

Scores of QUADAS-2 [7] evaluation are presented in Fig. 2. Overall, the studies showed a low-to-moderate risk of bias and a few concerns about applicability. Four studies scored low risk of bias in all domains of the QUADAS-2 system. The highest risk of bias was associated to flow and timing. Considering concerns regarding applicability, all studies but two presented a low risk.

### Post-operative complications rate

All studies reported the rate of patients who developed complications after rTAR or oTAR, with an overall rate of 17.4%.

**Fig. 3:**
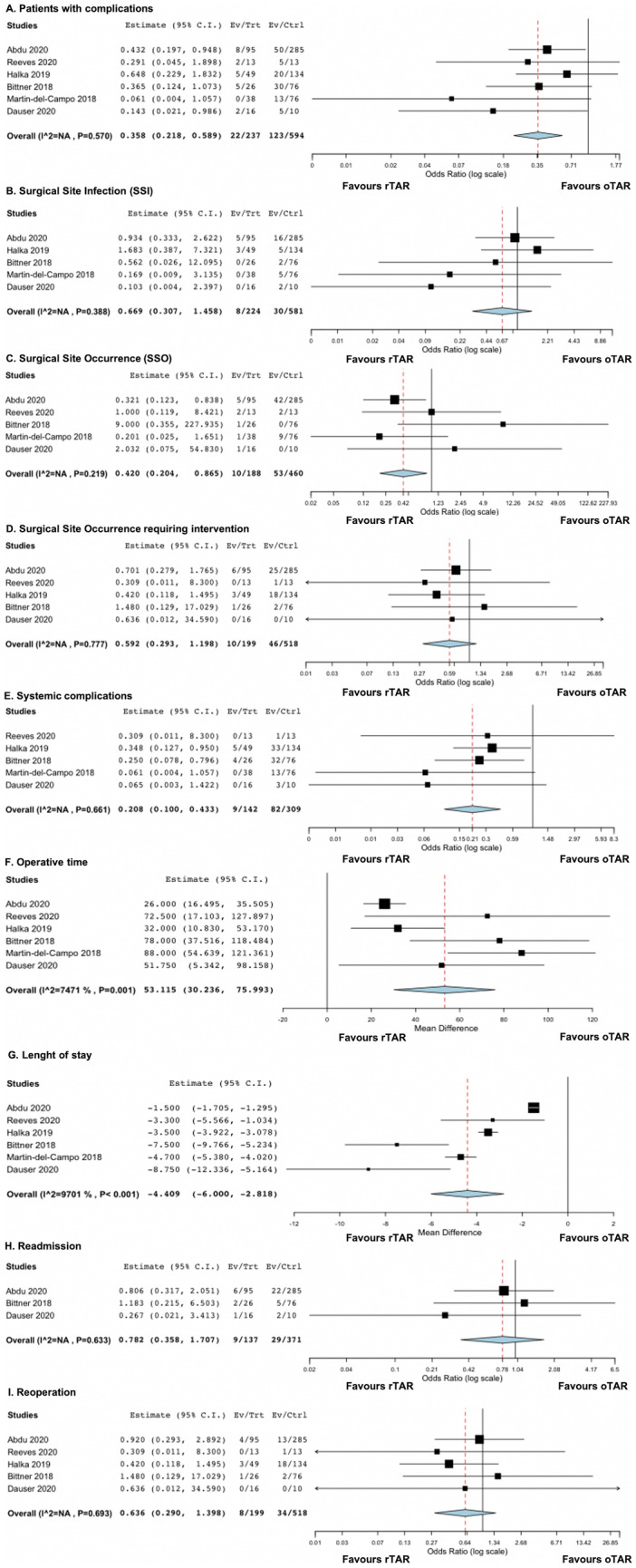
Forest plots of outcomes included in the analysis: patients with complications (**A**); SSI (**B**); SSO (**C**); SSO requiring intervention (**D**); systemic complications (**E**); operative time (**F**); length of stay (**G**); readmission (**H**); reoperation (**I**)

However, rTAR was associated with lower risk of complications rate (9.3 vs 20.7%, OR 0.358, 95% CI 0.218–0.589, *p* < 0.001) than oTAR (Fig. 3A). No heterogeneity was found in this analysis (I2 = 38.4%, *p* = 0.57).

Then, we performed a meta-regression to evaluate possible patient (age, gender, comorbidities such as diabetes, BMI, respiratory diseases) or disease (reason for stoma creation) or technical (stoma type, site of mesh placement) variables able to impact upon the outcomes.

At meta-regression, no variable impacted the risk of complications (age *p* = 0.52; gender male vs female *p* = 0.38; diabetes *p* = 0.31; BMI *p* = 0.23; respiratory diseases *p* = 0.28; ASA status *p* = 0.34; type of hernia *p* = 0.41; hernia dimensions *p* = 0.16, mesh used yes vs not *p* = 0.27, type of mesh synthetic vs hybrid vs biologic *p* = 0.31). The meta-regression found also that the length of follow-up was not able to influence the post-operative complications rate (*p* = 0.46).

### Surgical site infection

When we explored the surgical site infection (SSI) rate, we found 5 studies [8–12] on 805 patients reporting such outcome. The cumulative SSI rate was, therefore, 4.7%. There was no statistically significant difference in terms of risk of SSI development between rTAR and oTAR (3.6 vs 5.2%, OR 0.669, 95% CI 0.307–1.458, *p* = 0.44) (Fig. 3B). Also, in this case, there was no heterogeneity (I2 = 21%, *p* = 0.39).

### Surgical site occurrence

Five studies [8–10, 12, 13] involving 648 patients reporting the rate of SSO, with a cumulative rate was 9.7%. Robotic TAR was associated with lower risk of developing SSO compared with oTAR (5.3 vs 11.5%, OR 0.669, 95% CI 0.307–1.458, *p* = 0.02) (Fig. 3C). No heterogeneity was found in this analysis (I2 = 0%, *p* = 0.2).

However, when we evaluated the rate of SSOPI (five studies [8–11, 13] on 717 subjects), no differences were noted between rTAR and oTAR (5 vs 8.9%, OR 0.592, 95% CI 0.293–1.198, *p* = 0.11) (Fig. 3D). Also in this case, no heterogeneity was found (I2 = 0%, *p* = 0.78).

### Systemic complications

All studies but one [9–13], reported the rate of systemic complications after surgery (451 patients). The overall rate was 20.2%. However, rTAR was associated with lower risk of developing systemic complications as compared with oTAR (6.3 vs 26.5%, OR 0.208, 95% CI 0.100–0.433, *p* < 0.001) (Fig. 3E). No heterogeneity was found in this analysis (I2 = 0%, *p* = 0.7).

### Operative time

All studies reporting operative time differences between rTAR and oTAR. As expected, a significant longer operative time was reported in the rTAR group when compared with oTAR group, (SMD 53.115, 95% CI 30.236–75.993, *p* < 0.01) (Fig. 3F). High heterogeneity was found (I2 = 74.71%, *p* = 0.001).

### Length of hospital stay

The LOS was reported by all studies. Robotic TAR was associated with a significant shorter hospital stay than oTAR (SMD − 4.409, 95% CI − 6.000 to − 2.818, *p* < 0.001) (Fig. 3G). Overall heterogeneity was high in this analysis (I2 = 97%, *p* < 0.001).

### Readmission and reoperation

The need for readmission was reported by only three studies [8–10] and 508 patients. No differences were seen between rTAR and oTAR in terms of readmission rate (6.6 vs 7.8%, OR 0.782, 95% CI 0.358–1.707, *p* = 0.77) (Fig. 3H). No heterogeneity was found (I2 = 12.5%, *p* = 0.63). Similarly, the rate of reoperation, explored by 5 studies [8–11, 13] and 717 patients, was not significantly different between rTAR and oTAR (4% vs 6.5%, OR 0.636, 95% CI 0.29–1.398, *p* = 0.26) (Fig. 3I). No heterogeneity was found (I2 = 0%, *p* = 0.69).

## Discussion

In the current study, rTAR seems to be associated with lower risk of complications rate, with a significant longer operative time and a shorter hospital stay than oTAR. No difference was found about readmission and reoperation rate.

The oTAR with mesh reinforcement has emerged as an adequate approach for a VHR. It allows wide prosthesis overlap of larger hernia defects in the preperitoneal space lateral to the posterior rectus sheath. Ventral hernias with substantial width (≥ 10 cm) on cross-sectional imaging will often require TAR, and smaller defects may require TAR depending on the chronicity of the hernia and retraction of the rectus muscles. More generally, any patient who undergoes retrorectus dissection in whom the posterior sheath cannot be closed without tension likely needs unilateral or bilateral TAR [14]. It also seems to decrease the wound morbidity typically associated with the anterior component separation that requires the creation of a large subcutaneous flap [15, 16].

Meanwhile, to combine the benefits of minimally invasive approach and posterior component separation, rTAR is increasingly being used to treat complex ventral hernias. Some recent studies suggested that robotic approach seems to have some benefits despite a hypothetical increase of health-system costs [10].

From our review, all the analyzed papers reported a very low conversion rate (0–3%) suggesting that, in experienced hands, the robotic approach could be a feasible approach.

About post-operative complications, we found an overall rate of 17.4% after rTAR or oTAR, comparable with the international literature trend [17]. More particularly, we found a lower risk of complications rate in the rTAR group than the oTAR one (*p* < 0.001). This result is due to a lower risk of developing SSO and systemic complications after rTAR. No difference was found about SSI and SSOPI. Our results are consistent with that of a previous qualitative review about more generally robotic retromuscular repair. The authors reported a range of 3–52% of complications that are not necessarily infected (i.e., hematoma, seroma) [18]. This finding is not related to a proportional occurrence of SSOPI between both groups. Few papers reported a SSOPI rate ranged from 4 to 6%, for the most part reported as interventional radiology guided drainage [9, 16, 19, 20]. Few authors reported specific details regarding the nature of post-operative complications and there appeared to be broad distribution over various organ systems. No studies reported hernia recurrences with a length of follow-up ranged from 23 to 180 days.

These encouraging results could partly justify a decreased LOS for the rTAR group observed in our metanalysis. However, analyzing each metanalyzed papers, seems to be clear that the literature supporting robotic TAR is frankly lacking by some selection bias.

Martin-del-Campo et al. [12] which showed a shorter LOS with robotic TAR (1.3 days vs 6 days), reported more than double the number of patients with recurrent hernias in the oTAR group compared to the rTAR group (64.5 vs 28.9%, *p* = 0.001). Also Bittner et al. showed similar differences about the LOS (3.5 vs 6.7 days, *p* = 0.001). However, in the oTAR group, there was a 16% rate of concomitant procedures performed, including small bowel, colon, pancreatic, and kidney resections [9].

The first paper reporting a shortened LOS in the rTAR group was that of Carbonell et al. [16]. They suggested that this result may be attributable to decreased pain due to smaller incisions size in the rTAR versus oTAR group as well as a decreased traction on the abdominal wall by retractors.

The decreased LOS seems to be attributable to the typical advantages of a minimally invasive surgery respect to an open approach: reduced morbidity and less abdominal wall trauma seem to be the most important factors positively affecting this outcome.

Conversely, we found a longer operative time in the rTAR than oTAR group with a mean difference of about 53 min between the two surgical approaches.

This outcome is often affected when we compare a robotic procedure respect to an open one. This is due clearly to the docking and undocking steps required in the robotic operations that need additional time not required with open procedures. However, it cannot be excluded that this outcome may be affected also by cases performed during a learning curve period as reported by some study concerning robotic abdominal wall reconstruction (AWR) and other minimally invasive procedures [8, 16, 21, 22]. Abdu et al. [8] reported that the dataset timeframe includes cases from the beginning robotic experience of the two largest contributing authors to the robotic cohort. This may reflect, partly, increased time due to both surgeon techniques and case flow learning curve. The remaining 12 cases came from eight additional surgeons, for whom we would also expect longer operative times given they were in their experience with this technique.

However, Muysoms et al. showed that during the transition to robotic groin hernia repair there is a decrease in operative time by approximately 25% over a 4-month period of using the technology [23].

The higher cost of a robotic procedure represents always a discussed issue [24]. However, the cost analysis regarding the use of a robotic platform for AWR represents an item likewise debated. If we analyze the procedure-related, Personnel, reusable and disposables costs, we may of course conclude that a robotic AWR is not a cost saving procedure comparing to open AWR. But, as reported by Dauser et al. [10], adding procedure-related costs and costs for in-patient stay, total costs of EUR 8108.93 and 8650.12 were calculated for robotic-assisted and open TAR, respectively. However, the high costs for robotic AWR procedures result in an important issue about the economically sustainability of many National or Regional Health Systems.

Generally, the benchmark for a cost analysis of a robotic approach for quite all the digestive procedures, is represented by a laparoscopic approach. In the AWR, the robotic benchmark for a cost-analysis is the open approach because of the wide limitations found with the use of a laparoscopic approach during a complex AWR. So, it is conceivable that the use of a robotic platform for complex AWR may be comparable in total and social costs to the Open AWR. This is even more possible in the next future with a desirable reusables and disposables cost reduction due to the advent of new robotic platform on the health market.

The major strengths of this study are the systematic approach and that it is the first meta-analysis published on the direct comparison of the results of rTAR and oTAR.

Our study has some limitations. The retrospective nature of the studies analyzed is the main source of bias. None of them is a prospective randomized trial providing high level of evidence. The quality of the papers’ evidence was moderate, but proper blinding, for example, was always lacking. Accordingly, a selection bias was introduced.

These studies have no adequate follow-up period and so it is not possible to gain firm conclusions regarding the effectiveness of rTAR on recurrence rate. Two of the 6 six included studies [8, 11] report the results of Hybrid rTAR which could be another factor affecting the selection bias. Finally, none of the included studies clearly reported if the enrolled ventral hernia were PVH or IH. In a previous metanalysis [25], we supported the hypothesis that PVH and IH are different conditions with the latter being more challenging to treat. Accordingly, EHS classifications should be adopted systematically, as well as pooling data analysis should be no longer performed in clinical trials.

## Conclusion

Based on the data from our meta-analysis, robotic approach for TAR seems safe and fasible, even in more difficult cases. The rTAR shows the common advantages of minimally invasive procedures that improve short-term outcomes with significant benefits in the early postoperative period. However, no firm recommendations can be drawn from the available evidence and further prospective, randomized studies should investigate clinical outcomes with longer follow-up and perform comparative cost-effective analyses.
